# Supplementary material for: Exploring the associations between narcissism, intentions towards infidelity, and relationship satisfaction: Attachment styles as a moderator
Source: PLoS One. 2020 Nov 13;15(11):e0242277. doi: 10.1371/journal.pone.0242277 (PMC7665789; doi:10.1371/journal.pone.0242277)
Supplement: Attachment — Submitted filename: Response to Reviewers.docx [file pone.0242277.s003.docx]

**Response to Reviewers**

**We would like to thank each of the reviewers for their invaluable and very useful feedback & comments. We have completed the amendments on the manuscript with the comments of the reviewers and submitted the work to the journal once again. Below are the changes made on the manuscript:**

- **The repetitive places in the introduction part have been taken out of the work to present it more succinctly**
- **The use of Acronyms in Tables has been avoided. We used the full version of the words to make the Tables more easily understandable.**
- **Instead of presenting the manuscript as a two-study paper, we converted it into a single study with tests of mediation on a subgroup of the sample.**
- **We made changes in the references considering APA 7^th^ style**
- **We add justification for mediation.**
- **Introduction was shortened.**
- **Discussion was shortened.**
